# Insight from International Guidelines: do We Have Satisfactory Recommendations for Secondary Mitral Regurgitation?

**DOI:** 10.31083/j.rcm2305180

**Published:** 2022-05-17

**Authors:** Francesco Nappi, Sanjeet Singh Avtaar Singh, Antonio Fiore, Omar Ellouze

**Affiliations:** ^1^Department of Cardiac Surgery, Centre Cardiologique du Nord, 93200 Saint-Denis, France; ^2^Department of Cardiothoracic Surgery, Golden Jubilee National Hospital, G81 4DY Glasgow, UK; ^3^Department of Cardiac Surgery, Hôpitaux Universitaires Henri Mondor, Assistance Publique-Hôpitaux de Paris, 94000 Creteil, France; ^4^Department of Anesthesia, Centre Cardiologique du Nord, 93200 Saint-Denis, France

**Keywords:** secondary mitral regurgitation, international guidelines, mitral valve surgery, transcatheter edge to edge valve repair

## Abstract

Both the European Society of Cardiology (ESC) and the American College of Cardiology (ACC/AHA) have recently released guidelines on the management of patients with secondary mitral regurgitation. This includes defining, classifying, and assessing the severity of secondary mitral regurgitation. These guidelines are also the first to incorporate the use of transcatheter edge-to-edge repair in decision-making based on recent studies. The review highlights the strengths and shortcomings of these studies and the applicability and generalisability of these results to assist in decision-making for the heart time. It also emphasises the importance of shared decision-making via the heart team. Echocardiography plays an important role in the assessment of these patients although these may be specifically for primary mitral insufficiency. The optimal guideline-directed medical therapy should be the first line of treatment followed by mechanical intervention. The choice of intervention is best directed by a specialist multidisciplinary team. Concomitant revascularization should be performed in a subgroup of patients with severe secondary mitral regurgitation given the role of adverse LV remodelling in propagation of the dynamic secondary MR. The guidelines need further confirmation from high-quality studies in the near future to decision-making towards either TEER, mitral valve replacement, or mitral valve repair with or without a subvalvular procedure.

## 1. Introduction

The most important international guidelines and recommendations of the professional society of the European Society of Cardiology (ESC) [1] and the American College of Cardiology/American Heart Association (ACC/AHA) regarding the management of patients with secondary mitral regurgitation [2] were updated in 2020 and 2021. In the previous 2017 guidelines, there were notable differences in the recommendations regarding the management of patients with SMR, as well as new evidence that became available after the publication of the new guidelines led to a substantial change in the recommendations for the treatment of SMR [3, 4].

The objective of this review is to compare current ACC/AHA and ESC guideline recommendations regarding the management of patients with secondary mitral regurgitation. This review suggests an exploration into the differences between the 2 guidelines that summarize new data thus addressing these domains of discordance.

## 2. Decision Pathway from the Working Group of the International Guidelines.

Since 2014, international guidelines have established a presidential task force within the ACC/AHA and ESC to review clinical documents. The main recommendation of the Task Forces in drafting the guidelines was directed towards particular attention to concise decision paths and/or key points of care, which have replaced the more traditional paths based on the examination of longer documents [5, 6, 7].

Both Task Forces (i.e., ACC/AHA and ESC) have also focused their work on establishing new criteria for identifying clinical topics of relevant value to be addressed. An innovative approach was aimed at summarizing the elaborated contribution of the various interested components through systematic reflections meetings in the round table. This way of proceeding has led to the outlining of short and defined decision-making paths on key points with the result that the Expert Consensus Documents have been renamed “Expert Consensus Decision Paths” (ECDP) [8, 9, 10].

New data and experiences were accumulated leading to the reach for new ECDPs thus updating the previous ones, elaborated by the working groups of the international guidelines. The recommendations dictated by the international guidelines for valvular heart disease (VHD) were influenced by emerging evidence.

Higher-income country have a higher incidence of the degenerative aetiology of mitral regurgitation (MV). Of these, a large percentage are elderly people with multiple comorbidities. At the same time, new definitions of the severity of SMR appear in the guidelines, supported by the results of randomized studies performed on this patient population. In addition to degenerative and ischemic aetiology, patients with rheumatic disease are still recruited and studied in less industrialized countries [1, 2].

The identification and definition of mitral valve regurgitation (MVR) is primarily based on the use of echocardiography which is established as the key technique for diagnosing VHD and assessing its severity and prognosis. In support of this investigative approach, other non-invasive imaging methods have affirmed their increasingly important role such as cardiac computed tomography, cardiac magnetic resonance, and biomarkers play a more central role [10, 11, 12, 13, 14, 15, 16, 17, 18].

The defined assessment of the patient and the indication for treatment are provided by the multidisciplinary centers for heart valves and the referral centers for the treatment of heart valve diseases. The evaluation of the heart teams (HT) is considered relevant for an evaluation centered on the type of intervention to be proposed to the patient. The role played by the HT takes into account both the new platforms for the treatment of structural heart diseases and taking into account the expectations and values of the patient [10, 11, 12].

The emergence of new approaches focussing on the transcatheter techniques are strongly supported by the results published in randomized controlled trials (RCTs) which have compared the new, less invasive transcatheter procedures with standard surgical approach, contributing to the diversion from previous guidelines. The new guidelines are responsible for clarifying the role of each procedure in low-risk patients. This aspect is clearly evident for patients receiving a transcatheter mitral procedure. The new international guidelines have included edge-to-edge transcatheter repair (TEER) in the recommendations for the treatment of SMR as an alternative to optimal medical therapy for patients who meet specific criteria. Likewise, the increased number of studies on transcatheter valve implantation in the valve after the failure of surgical bioprostheses has led to its updated indications [1, 2, 19, 20, 21, 22, 23, 24, 25, 26, 27, 28, 29, 30, 31, 32, 33, 34].

**Fig. 1.**
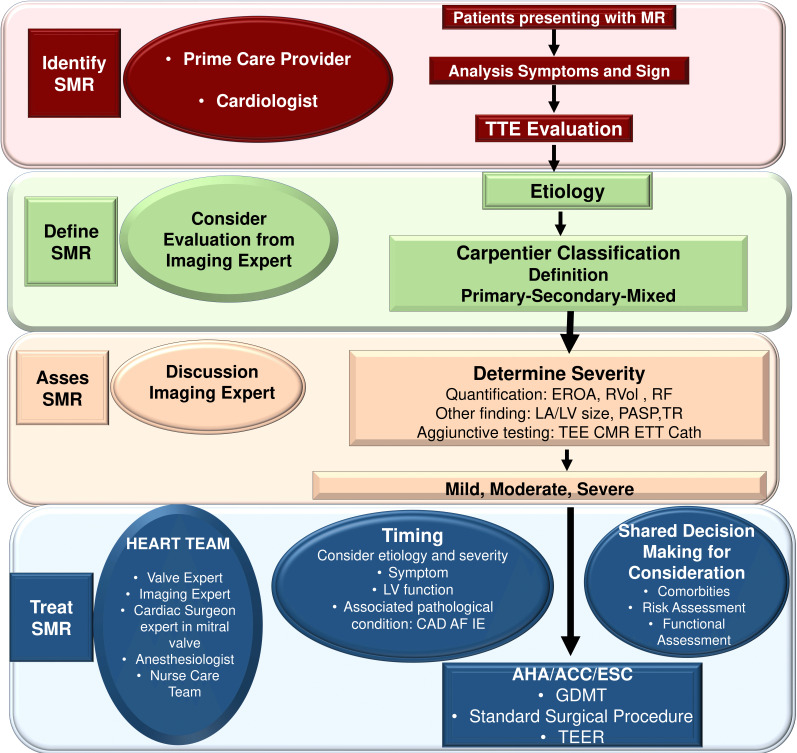
**provides an overview of the different steps to follow in the decision-making process which include identification, definition, assessment and treatment strategy for SMR**. Abbreviation: AHA, American Heart Association; ACC, American College of cardiology; AF, atrial fibrillation; CAD, coronary artery disease; Cath; catheterism; CMR, cardiac magnetic resonance; EROA, effective regurgitant orifice area; ESC, European Society of Cardiology; GDMT, guideline-directed medical therapy; IE, infective endocarditis; LA, left atrium; LV, left ventricle; PAPS, pulmonary artery systolic pressure; RF, regurgitant fraction; RVol, regurgitant volume; SMR, secondary mitral regurgitation; TEE, transoesophageal echocardiography, TEER, transcatheter edge to edge repair; TR, tricuspid regurgitation. From Vahanian, *et al*. [1]; Otto CM, *et al*. [2]; Iung B, *et al*. [11]; Nishimura RA, *et al*. [14]; Chambers JB, *et al*. [34].

**Fig. 2.**
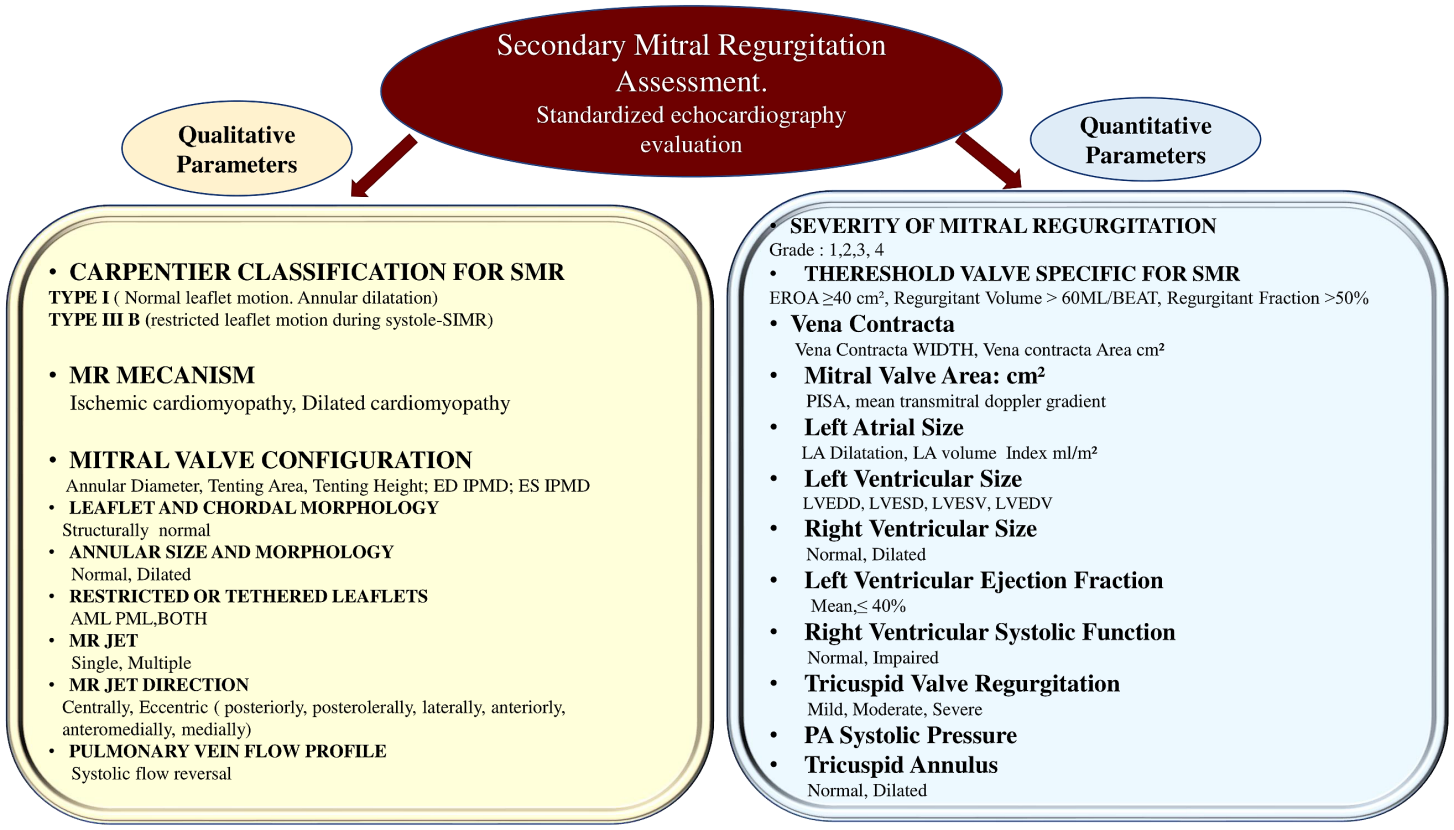
**Qualitative and Quantitative Parameters for Standardized Echo Reporting in patient with SMR**. Abbreviations in other figures. From Baumgartner H, *et al*. [10]; Zoghbi WA, *et al*. [15]; Zoghbi WA, *et al*. [16]; Chambers JB, *et al*. [34].

Fig. 1 (Ref. [1, 2, 11, 14, 34]) shows the different steps to follow in the decision-making process which include identification, definition, assessment, and treatment strategy for SMR. The descriptors of SMR mechanism and severity that should be included in standardized echocardiographic reports are listed in Fig. 2 (Ref. [10, 15, 16, 34]).

One of the main roles of the writing groups of International Guidelines, through the Expert Consensus Documents, is to develop more usable algorithms, which have accelerated the delivery of directions and recommendations to points of care. For example, the decision-making paths of the American and European guidelines are not intended to provide a single correct answer. It is primarily aimed at encouraging physicians to ask definitive questions and examine relevant factors before making recommendations and discussions with the patient. As multiple paths can be taken for treatment options, the guidelines also help doctors make a more informed decision [8, 9].

In the current scenario of the cardiological sciences, there are definite advances in the field of multimodal imaging, surgical techniques, and results. In addition, with the introduction of valve replacement and repair using the transcatheter technique, a substantial paradigm shift has transformed the approach to patients with structural heart disease. Reports noting long-term survival in patients who have been treated for structural heart valve disease have certainly guided the clinical decision-making process regarding the appropriate timing for valve interventions [10, 11, 12, 13].

Although it has given a large contribution to the scientific literature, currently, there are gaps in knowledge and performance that can negatively influence the postprocedural outcomes in patients. From this perspective, the tools of practice need substantial means of improvement. A case in point can be found in the assessment and management of patients with mitral regurgitation (MR), a highly prevalent disease among the elderly in the United States and Europe. The diagnostic classifications of these patients are more complex, partly linked to the various causes, dynamic nature, and insidious progression. MR results from functional impairment or anatomical disequilibrium that involves one or more elements of the mitral apparatus required for usual regular function, including the left ventricle, papillary muscles, chordae tendinae, leaflets, and annulus [14, 15, 16, 17, 18].

The International Guidelines contain consensus recommendations of clinical experts to guide the approach to patients identified with rare diseases. The documents elaborated underline clinical and echocardiographic evaluations, the etiology, and the pathoanatomical mechanism. The choice of treatment to use also takes into consideration the haemodynamic consequences derived from the valvular pathology, the recognition of precipitating clinical conditions that require referral surgery, estimation of the difficulty to perform a mitral valve repair through the evaluation of pathanatomy, and understanding the present role devoted to the mitral transcatheter edge-to-edge repair (TEER) in the United States and Europe [19, 20, 21, 22, 23, 24, 25, 26, 27, 28, 29].

## 3. Take-Home Messages for the Treatment of Secondary Mitral Regurgitation in Exploring International Guidelines. The Role of Heart Valve Center and Heart Team

In the evaluation of patients with secondary mitral regurgitation who are suitable for an interventional approach, the role of a multidisciplinary heart team (HT) as either the referral centre or for a consultation is aimed at ensuring a complete evaluation and a bespoke choice of procedural modality. Two primary characteristics are identified for a recognized Heart Valve centre (HVC). Firstly, carrying out continuous professional training towards a targeted clinical subspecialty interest. Secondly, the HVC as the referral centre for the treatment of heart valve diseases, and should promote the timely referral of patients with VHD for complete evaluation before irreversible harmful progression of cardiac disease occurs. The status of HVC for the treatment of SMR is achieved through a process that includes a high volume of procedures performed coupled with a high level of competence and specialized professional training [30, 31, 32, 33, 34].

Current evidence has reinforced the crucial role of the Heart Team, which should summarize the clinical, anatomical, and procedural features by integrating them with conventional scores and with informed the patient’s treatment of choice. Therefore, all decisions regarding treatment and intervention should be made by a homogeneous HT with experience in VHD that is inclusive of clinical and interventional cardiologists, cardiac surgeons, imaging specialists with experience in interventional imaging [31, 32], cardiovascular anaesthesiologists, and other specialists if necessary (e.g., heart failure specialists or electrophysiologists). For management of the patient with secondary mitral regurgitation, in addition to the skills necessary for the management of valve interventions, expertise in the interventional and surgical management of coronary artery disease (CAD), vascular disease and complications must be present [34, 35, 36, 37].

While carrying out the work of the heart team, history and physical examination findings should be correlated with the results of noninvasive testing. An even greater impetus is given to non-invasive evaluation using three-dimensional (3D) echocardiography, cardiac computed tomography (CCT), cardiac magnetic resonance (CMR), and biomarkers. In fact, these methods have played an increasingly central role. However, the different steps require rigorous application. Therefore, if there is a discrepancy between physical examination and initial non-invasive tests, additional non-invasive (computed tomography, cardiac magnetic resonance, stress test) or invasive (transoesophageal echocardiography, cardiac catheterization) tests should be considered to determine the optimal treatment strategy [15, 16, 18, 22, 24, 25].

**Fig. 3.**
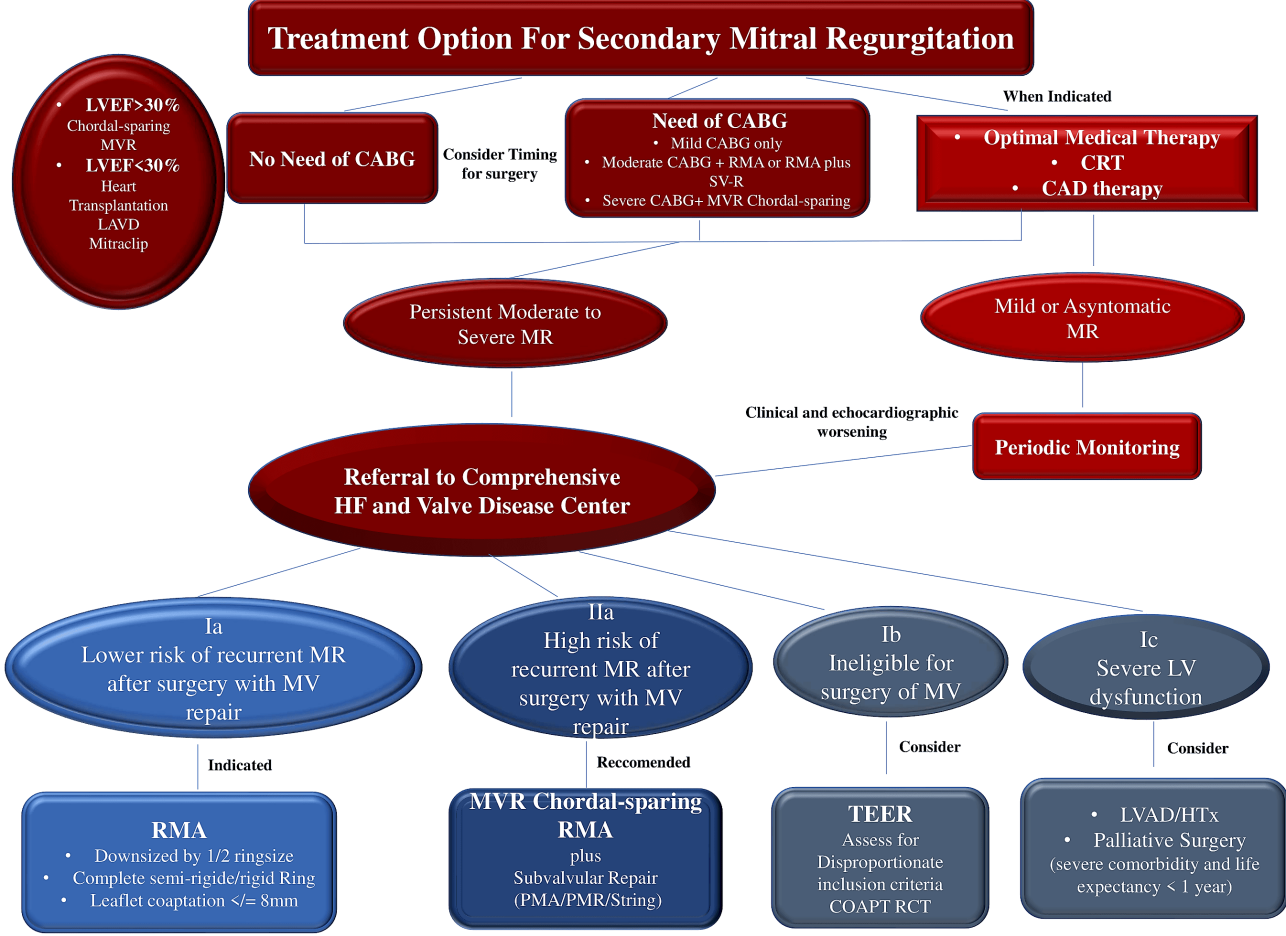
**Treatment option for secondary mitral regurgitation**. Abbreviation: CABG, coronary artery bypass grafting; CRT, cardiac resynchronization; HF, heart failure; LAVD, left ventricle assistance device; LV, left ventricle; LVEF, left ventricular ejection fraction; MR, mitral regurgitation; MV, mitral valve MVR, mitral valve repair; RMA, restrictive mitral annuloplastie; SVR, subannular repair; TEER, transcatheter edge to edge repair. Others abbreviation in Fig. 1. From Vahanian, *et al*. [1]; Otto CM, *et al*. [2]; Stone GW, *et al*. [23]; Petrus AHJ, *et al*. [39]; Harmel EK, *et al*. [40]; Nappi F, *et al*. [41]; Acker MA, *et al*. [42]; Obadia JF, *et al*. [43]; Iung B, *et al*. [44].

Recently, mitral transcatheter edge-to-edge repair (TEER) has proved beneficial in a selected subset of patients with secondary mitral regurgitation who remain severely symptomatic despite guideline-directed management and therapy for heart failure. Screening the new international guidelines, it emerged that TEER is increasingly used in SMR and has been evaluated against optimal medical therapy resulting in a noticeable increase in recommendation [19, 20, 21, 23, 26, 27, 38] (Fig. 3, Ref. [1, 2, 23, 39, 40, 41, 42, 43, 44]).

Finally, particular attention was given by the 2021 ESC guidelines to the new definitions of the severity of secondary mitral regurgitation (SMR) based on the outcomes of studies on interventions that can be discussed by the heart team. In fact, contrary to the 2017 guidelines, a new section with the indications for mitral valve intervention in chronic severe secondary mitral regurgitation has been added to the 2021 guidelines [1, 2, 3, 8].

## 4. Evaluation of Class of Recommendation and Level of Evidence

Very scarce recommendations for secondary mitral regurgitation are based on Level of Evidence (LOE) A in ACC/AHA the guidelines [2] with the exception of one recommendation concerning the medical therapy. Most of these are graded as level of evidence (LOE) B. This implies that the guideline writing group essentially worked by analyzing moderate-quality evidence from 1 or more randomized clinical trials or RCT meta-analyses (LOE-BR) or moderate-quality evidence from well-designed non-randomized observational cohort studies or registry studies, as well as meta-analysis of these studies (B-NR). Instead, the few LOE A emerged in the recommendations for the treatment of SMR is because the ACC/AHA writing group assessments were not based on high levels of evidence from more than 1 RCT, meta-analysis of high quality of RCTs and one or more RCTs corroborate by the high quality of registry studies [2].

**Fig. 4.**
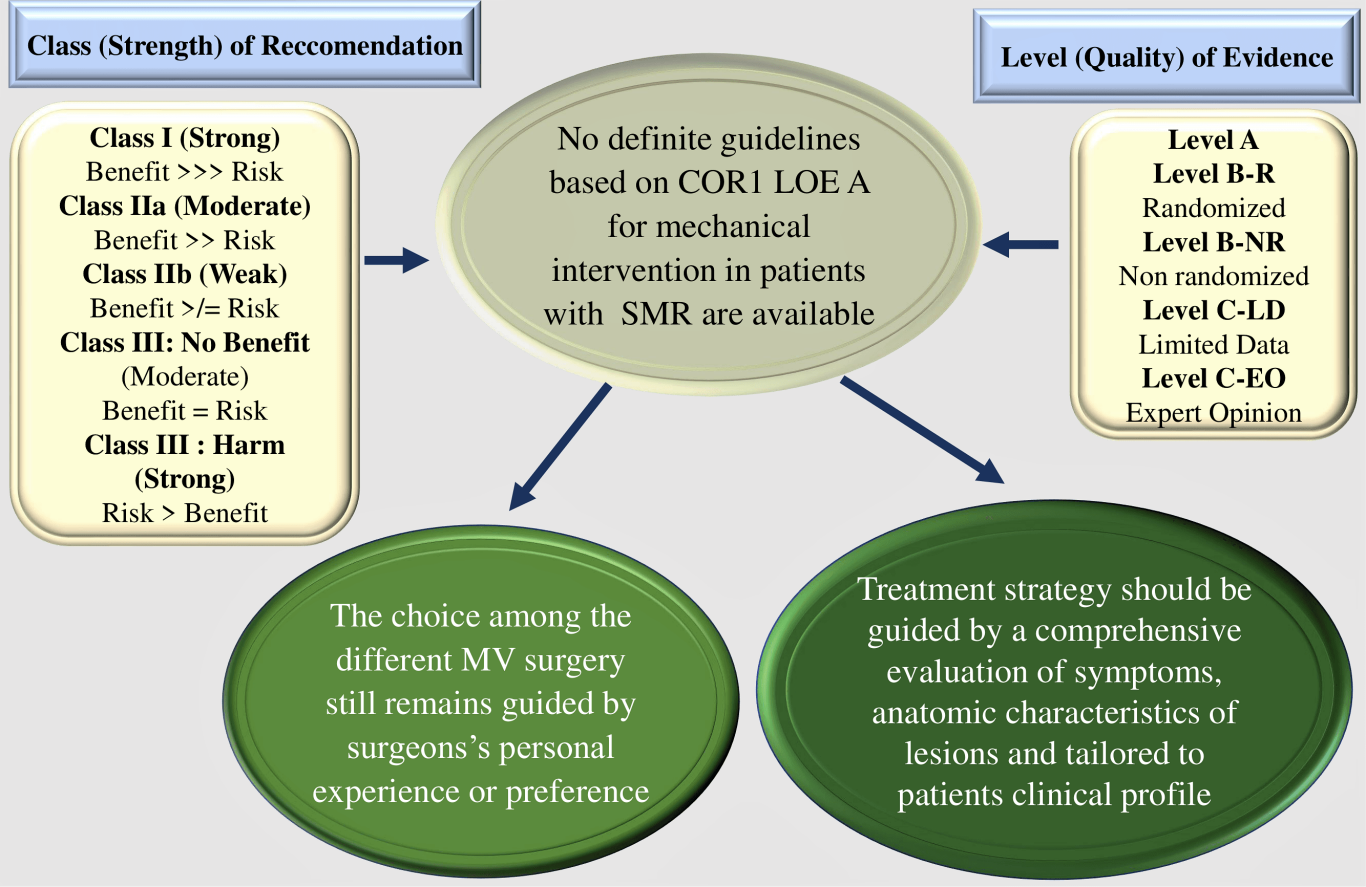
**Class of recommendation (COR) and the level of evidence (LOE) that direct the degree of choice for a treatment**. 2020 ACC/AHA guidelines and 2021 ESC guidelines elaborated very scarce recommendations for secondary mitral regurgitation that are based on Classe of Recommandation 1 (COR 1) and Level of Evidence (LOE) A in ACC/AHA and the guidelines. Abbreviations: COR, Classe of Recommandation; LOE, Level of Evidence; SMR, secondary mitral regurgitation. From Vahanian, *et al*. [1]; Otto CM, *et al*. [2].

In 2021 ESC guidelines [1] no recommendations graded with COR 1 LOE A was reported. Of a total of six recommendations 50% were graded as Class IIa or IIb and Level of Evidence C. Therefore, there is conflicting evidence and/or divergence of opinion about the usefulness or efficacy of the given treatment or procedure emerging from the reports. In patients with SMR classified as Class IIa, a standard surgical approach or TEER should be considered because the weight of evidence or opinion is in favour of the usefulness or efficacy of mechanical intervention. Conversely, in patients graded as Class IIb, the intervention may be considered and the utility or effectiveness of mechanical intervention is less well determined by evidence or specific advice. In this population, the usefulness or effectiveness determined by TEER is unknown, unclear, uncertain, and not well established. Therefore, the writing investigators worked on the limited data that has been reported in a single randomized clinical trial or large non-randomized studies as well as on consensus opinions of experts and/or small studies, retrospective studies, and registries [1] (Fig. 4, Ref. [1, 2]).

## 5. Echocardiographic Assessment: the General Principle

Echocardiography is the cornerstone of mitral regurgitation (MR) diagnosis. It allows evaluation of mechanism: primary or secondary, etiology and severity of MR. Moreover, echocardiography allows the establishment of a reference point for MR follow-up or surgical and transcatheter procedure guidance [10, 11, 15, 16]. Diagnosis and assessment of mitral regurgitation severity requires a multiparametric approach. Transesophageal echocardiography is complementary to transthoracic echocardiography, particularly if the image quality in transthoracic approaches are poor. Moreover, transesophageal echocardiography allows better evaluation of MR mechanism and etiology, and recent integration of three-dimensional (3D) echocardiography undoubtedly offers the finest evaluation of the mitral valve and better guidance of surgical or transcatheter interventions [10, 31, 32, 33, 34]. Echocardiographic assessment of MR requires a dynamic approach, integrating loading conditions and volume status [15, 31, 45, 46, 47].

Echocardiographic quantification of mitral regurgitation severity has been the subject of several ESC [1, 10] and ACC/AHA [2, 15, 16] guidelines, but these mainly concern primary MR and the quantification of secondary mitral regurgitation from the parameters used to grade the severity of primary mitral regurgitation. The transposition of these parameters to SMR requires special precautions, and a multiparametric evaluation is mandatory. Recent ESC guidelines fill this gap with recommendations dedicated to SMR [16, 45] (Table 1 and Fig. 1).

**Table 1.** **Comparison of echographic criteria between European and American guidelines for quantification of severe secondary mitral regurgitation**.

	ESC Guidelines 2017	ESC Guidelines 2021	ACC/AHA Guidelines 2020
Qualitative			
Mitral valve morphology	No specific guidelines for secondary mitral regurgitation	Normal leaflets, severe tenting, poor leaflet coaptation	No specific guidelines for secondary mitral regurgitation
Colour flow jet area or jet area/left atrial area ratio		Large central jet (>50% of left atrium) or eccentric wall impinging jet of variable size	
Flow convergence		Large throughout systole	
Continous wave Doppler jet		Holosystolic/dense/triangular	
Semiquantitative			
Vena contracta width (mm)	No specific guidelines for secondary mitral regurgitation	≥7 mm (≥8 mm for biplane)	No specific guidelines for secondary mitral regurgitation
Pulmonary vein flow		Systolic flow reversal	
Mitral inflow velocity		E-wave dominant (>1.2 m/s)	
TVI mitral/TVI aortic		>1.4	
Quantitative			
Effective regurgitant orifice area (two-dimension PISA)	≥20 ${\mathrm{mm}}^{2}$	≥40 ${\mathrm{mm}}^{2}$ (may be ≥30 ${\mathrm{mm}}^{2}$ if elliptical regurgitant orifice area)	≥0.4 ${\mathrm{cm}}^{2}$
Regurgitant volume (mL/beat)	≥30 mL	≥60 mL (may be ≥ 45 mL if low flow conditions)	≥60 mL (lower in low flow states)
Regurgitant fraction (%)		≥50%	≥50%
Structural			
Left ventricle		Dilated	
Left atrium		Dilated	

ESC, European Society of Cardiology; ACC, American College of Cardiology; AHA, American Heart Association; TVI, time velocity integral; PISA, proximal isovelocity surface area.

The first step in assessing MR severity is the qualitative assessment to allow the diagnosis and classification to be made. Initial two-dimensional echocardiography will evaluate mitral valve morphology and rule out primary mitral regurgitation or a mixed etiology. The assessment of the mitral morphology will allow visualization of the mitral valve tenting and possibly calculate the tenting area alongside leaflet coaptation distance, length and visualize mitral leaflets thickening in addition to assessing for decreased mobility with systolic restriction (Carpentier Classification IIIb). It should be noted that morphological evaluation of the mitral valve during SMR is only evaluated in the recent ESC guidelines [1]. However, no threshold is proposed for the classification of severity according to tenting area or coaptation length. Only an overall assessment of the severity of the tenting is discussed. Mitral annular dilation is usually present with decreased annular contractility. Color Doppler evaluation is essential and will allow assessment of mitral regurgitation flow convergence, direction, and ratio with the left atrium. Semi-quantitative parameters include vena contracta width, assessment of pulmonary and mitral flow [10, 15, 31, 32]. The quantitative evaluation found in all the guidelines, whether American or European, classify the severity of mitral insufficiency according to the effective regurgitant orifice area (EROA) calculated by the proximal isovelocity surface area (PISA) method, the regurgitant volume, and the regurgitant fraction, which are largely dependent on left ventricular volumes and function [1, 2, 10, 15, 16] (Table 1 and Fig. 2).

3D echocardiography and particularly evaluation of 3D vena contracta is more accurate than EROA calculated by PISA in SMR. Indeed, EROA may be underestimated if the regurgitation orifice is elliptical rather than circular and in the case of multiple jets, which is a frequent condition in SMR. In this way, 3D vena contracta overcomes PISA limitation and particularly in SMR by directly calculating regurgitant orifice area [15, 48]. Moreover, 3D vena contracta is correlated with magnetic resonance imaging MR quantification. Cardiac function evaluation is mandatory in SMR quantification, particularly the left ventricle and atrium. A global (two-dimension and 3D, volume, dimensions, and ejection fraction) and regional left ventricle assessment will help diagnosis of SMR etiology [15, 16, 17, 18, 19, 20, 21, 22, 23, 24, 25, 26, 27, 28, 29, 30, 31, 32, 33, 34, 35, 36, 37, 38, 45, 47].

ACC/AHA and ESC guidelines differ in their classification of MR severity [1, 2]. ACC/AHA SMR severity is classified as stage A to D (asymptomatic to severe symptomatic) [1], while ESC guidelines classify MR as mild, moderate, and severe [2]. Moreover, there is a common gap between the 2017 ESC guidelines [3] and the 2020 ACC/AHA guidelines [2] concerning SMR. They both only include a quantitative evaluation based on the calculation of the EROA, regurgitant volume, and regurgitant fraction. In addition, the thresholds chosen for severe SMR diagnosis differ and are lower in the 2017 ESC guidelines [3] (EROA of 20 ${\mathrm{mm}}^{2}$, regurgitant volume of 30 mL) mainly linked to a worse prognosis associated with these lower thresholds in SMR. The 2021 ESC guidelines partially overcome these by adding specific guidelines for SMR classification [1]. Indeed, they provide extensive criteria encompassing qualitative evaluation of mitral valve morphology with leaflets tenting and left heart chambers assessment but without well-defined thresholds. Moreover, quantitative evaluation of SMR and particularly EROA, regurgitant volume, and regurgitant fraction evaluation join the ACC/AHA guidelines thresholds for severe SMR diagnosis (EROA of 40 ${\mathrm{mm}}^{2}$, regurgitant volume of 60 mL, and regurgitant fraction of 50%). Unfortunately, all of these guidelines still do not include a 3D assessment of the SMR severity, which is more accurate, due to the specific mechanism in SMR [1, 2, 3, 8, 16, 31, 32, 45, 46, 47].

### 5.1 Echocardiographic Assessment of MR Severity

#### 5.1.1 Color Flow Doppler Jet Size

Patients who experience severe mitral regurgitation are commonly evaluated with a TTE or TEE using Color Flow Doppler Jet Size (CFD). Concerns related to the echocardiographic image provided through CFD are because it is not a flow image, therefore only provides spatial distribution of the velocities within the image plane and is strictly dependent on the instrument settings and hemodynamic factors [15, 48]. The high-speed MRI jets occurring in patients with various pathologies such as aortic valve stenosis, LV outflow tract obstruction, or hypertension, lead to a misinterpretation of the severity of MR that appears worse on CFD [48].

Accurate recording of the blood pressure, the left ventricular systolic pressure estimated in the presence of aortic stenosis or obstruction of the left ventricular flow, heart rate, and rhythm are needed to affirm reliability at the time of performing the TTE assessment. All of these parameters must be integrated when assessing the severity of mitral regurgitation [10, 15, 31, 32, 45].

Uretsky *et al*. [49] recently reported the tendency of CFD to overestimate the severity of MR using cardiac magnetic resonance (CMR) compared to TTE which allows for more precise quantification of the jet. These results reinforced the findings of Singh *et al*. [50] who had previously observed that healthy individuals with no heart murmur often exhibited mild MR on CFD. On the other hand, a significant underestimation of MR is possible in patients in with low-velocity jets or markedly eccentric ones, causing the transfer of momentum to the LA wall [48]. Sahn *et al*. [51] revealed that in addition to jet guiding speed and eccentricity, also CFD jet size is affected by multiple other technical and hemodynamic factors. Therefore, based on this clinical and echocardiographic evidence, both US and European guidelines recommend not using the CFD-assessed MR jet size alone to assess MRI severity [15, 52].

#### 5.1.2 Quantitative Parameters

The calculation of EROA is crucial for the assessment of severity SMR as a marker of lesion severity associated with the determination of RVol and regurgitant fraction (RF) [15, 48]. These parameters can be measured through several methods, including the value of proximal isovelocity surface area (PISA), volumetric value, and 3-dimensional imaging value.

**Fig. 5.**
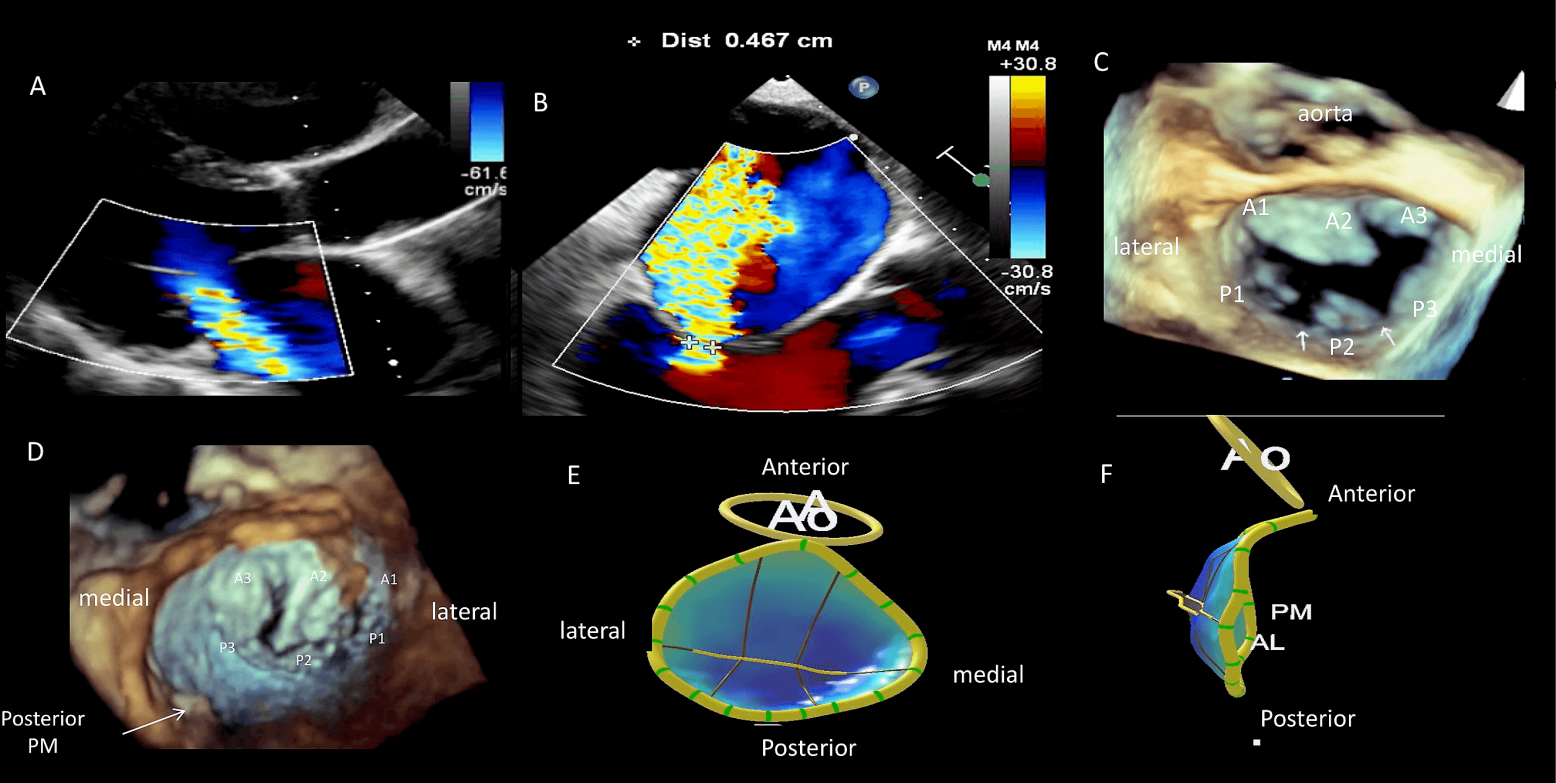
**Multimodality echocardiographic imaging of secondary ischemic mitral regurgitation**. A 70 y-old patient presenting with ischemic cardiomyopathy and LVEF at 25%. Three-vessel disease including right coronary occlusion has been diagnosed. TTE parasternal long axis view (A) and TEE LVOT view (B) show eccentric jet of MR due to asymmetrical tethering. (C) 3D TEE- en face view from LA showed marked indentations between P2-P3 and P2-P1 (white arrow) due to LV remodeling. (D) 3D TEE- en face view from LV showing an apical and posterior displacement of posterior papillary muscle (white arrow) secondary to localized LV remodeling. (E,F) MVQ software permits a reconstruction and modalisation of mitral valve, showing here a defect in the coaptation of mitral leaflets due to a tethering of the posterior valve induced by LV remodeling. Abbreviation: Ao, aortic; AL; anterior leaflet; A1,2,3 anterior scallop of MV; P1,2,3 posterior scallop of MV; PPM, posterior papillary muscle.

It is important to point out that all of these methods of measurement suffer from technical limitations and imprecision with substantial overlap of values recorded. First, the use of volumetric methods (including those with CMR) may be inaccurate in the multiplication of errors concerning the interpretation of the measurement of systolic volumes in certain locations. However, volumetric methods refer to the whole of mitral regurgitation throughout the duration of the systole. Second, the use of single-frame measurements including PISA or vena contracta width or area may lead to marked overestimation of MR severity in patients who present with a jet that is confined to early or late systole [53] (Fig. 5). Third, patients who have severe MR but have intermediate value measurements, cannot be classified. This is the case of patients with lower EROA and RVol values, which can underestimate the severity of the lesion. The secondary MR is associated with the characteristic morphology of the mitral valve orifice with crescent geometry that produces a falsely low PISA value for the respective EROA, due to its intrinsic supposition of a round orifice [54, 55, 56, 57, 58, 59, 60, 61]. Another example of underestimating lesion severity is the presence of multiple MR jets. In these patients, the EROA measured by a single jet does not reflect the totality of the MR [61, 62, 63]. Although the addition of multiple areas of EROA or vena contracta is reasonably accurate, this measurement has not been well validated [61, 62, 63].

Finally, in women, it is not uncommon to find lower quantitative values in the context of relatively smaller LV volumes. In such cases, severe MR is usually associated with other signs [57].

Several studies demonstrate that MR severity is dynamic [64, 65, 66]. Therefore, the results of chronic MR on LV and LA volumes and pulmonary artery pressure must be accounted for in a supplementary manner. It is common practice that the first approach performed by the doctors when they have to interpret an echocardiogram is to look at CFD to identify the presence of MR and to define its severity through a first impression. As shown in Fig. 4, although this evaluation constitutes a first starting point, it requires further confirmation that can be obtained using a Bayesian approach capable of integrating multiple factors to reach a final decision. Once the severity of the MR has been established at an initial evaluation, it should subsequently be determined whether the LA and LV dimensions are normal as well as to establish the holosystolic characteristic of the MR. The most fitting example can be represented by a patient in whom, at a first evaluation based on the presence of a large CFD jet, MR can manifest itself as severe. When the LA and LV dimensions are normal and MR is limited to late systole, the initial impression may be misleading towards probable overestimation.

These are the cases in which although the TEE may be sufficient to define the pathology of the leaflets and give a quantification of the severity of the MR; however, during this examination, the risk of underestimating the severity of MR during general anesthesia is possible and is due to favorable loading conditions. The valid alternative is represented by the use of CMR, which is a generally more accurate and reproducible procedure for quantifying RVol and RF as well as the determination of LV volumes and LVEF [15, 67, 68].

#### 5.1.3 Left Ventricular Global Longitudinal Strain

Two-dimensional left ventricular global longitudinal strain (LV GLS) is an interesting and evolving tool in secondary mitral regurgitation evaluation. Indeed, an altered LV GLS is independently associated with mortality in these patients [69]. LV GLS allows early screening and better disease severity classification, even before left ventricular ejection fraction (LVEF) alteration. Reduced LV GLS in patients with preserved LVEF was associated with a worse outcome after mitral valve surgery and lower post-operative LVEF [70]. LV GLS is a more sensitive tool in evaluating myocardial damage and fibrosis than LVEF, which can be overestimated in mitral regurgitation. Moreover, for patients undergoing mitral valve surgery or MitraClip device, lower baseline LV GLS can be associated with reduced ventricular remodeling after mitral regurgitation correction [71]. Similarly, reduced global peak atrial longitudinal strain predicts cardiovascular events in patients followed for mitral regurgitation [72]. Moreover, global peak atrial longitudinal strain is a useful prognostic marker of cardiovascular events in patients with moderate asymptomatic mitral regurgitation [73]. In these cases, reduced global peak atrial longitudinal strain can be a reliable index for earlier mitral valve surgery to improve outcomes.

#### 5.1.4 Integration Diagnostic Procedures 

Right and left heart catheterization may be useful to evaluate hemodynamics. Although limitations are dictated by the use of this approach, however with the performance of high-quality biplane LV angiogram additional information to work out diagnostic doubt can be provided. The assessment of invasive measurement of pressures, cardiac output, and pulmonary vascular resistance aims to facilitate a comprehensive judgment. Thus, the results that emerged can be tallied with clinical manifestations and with response to optimal medical treatment.

The additional use of stress echocardiography may also elucidate any discrepancies between noninvasive and clinical findings as well as help cardiologists to better elucidate MR severity, symptoms, exercise capacity, left/right ventricular responses to exercise, and pulmonary artery systolic pressure. High-quality CMR is extremely worthwhile in patients who have uncertain MR severity. However, this technology is only largely available in referral centers. For this reason, physicians may take into consideration referring such patients to a comprehensive valve center for multidisciplinary evaluation and treatment.

**Fig. 6.**
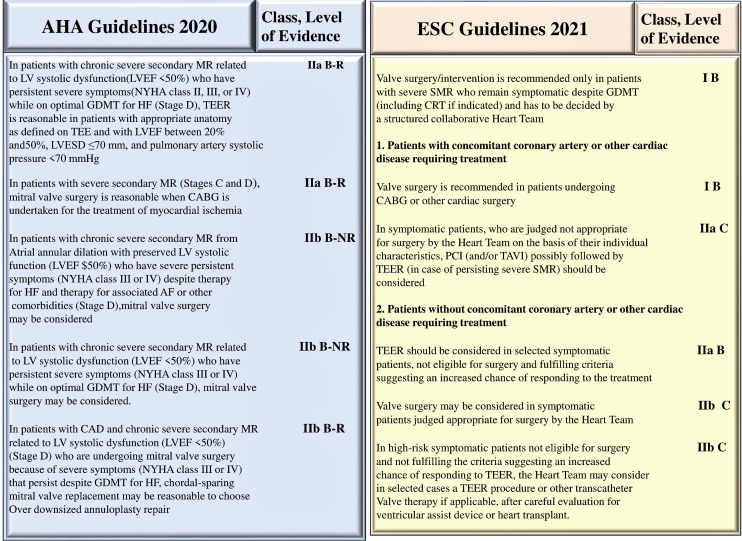
**Recommendations on indications for mitral valve intervention**. (Left) 2020 ACC/AHA guidelines. (Right) 2021 ESC guidelines. Abbreviation: PCI, percutaneous coronary intervention; TAVI, trancatheter aortic valve implantation. From Vahanian, *et al*. [1]; Otto CM, *et al*. [2]. Others abbreviation in Fig. 1.

An evidence-based algorithm for the assessment and management of patients with MR is delineated in Fig. 6 (Ref. [1, 2]). Based on the 2020 ACC/AHA Guideline for the Management of Patients with Valvular Heart Disease [2], this algorithm aims to alleviate any potential discrepancy related to the clinical approach in patients with MR [74]. Deciding when patients with MR should be referred for further clinical evaluation or valve intervention can be challenging. Once the clinical recognition of MR is determined by TTE, the next step is to ascertain in which clinical context does the pathology emerge. So the investigation about the symptomatology, the etiology of MR (primary vs. secondary vs. mixed), and the severity of MR are required with the use of the integrative methods previously delineated. With this algorithm, we aim to provide sequential steps for the clinician to can pilot a course toward decision making for additional analysis or referral for definitive treatment.

## 6. Recommendations from International Guidelines for the Management of Valvular Heart Disease

### 6.1 Medical Therapy

In patients with SMR, the first step in treatment is to establish the optimal medical therapy in compliance with the guidelines for the management of heart failure [75]. Today, with regards to the ESC guidelines, the most common option is the replacement of ACEI or ARB with sacubitril/valsartan. The use of sodium-glucose co-transporter 2 inhibitors and/or ivabradine, when indicated, represent another option of optimal therapeutic choice [76, 77].

ACC/AHA recommends (COR 1/LOE A) in patients included in stages C and D who experienced chronic severe secondary MR with HF and reduced LVEF the use of standard GDMT for HF, which is based on the administration of ACE inhibitors, ARBs, beta-blockers, aldosterone antagonists, and/or sacubitril/valsartan [74, 75, 76, 77, 78, 79, 80, 81, 82, 83]. The specified role is mandated to an expert cardiologist (COR 1/ LOE C-EO) for the management of patients with severe SMR presenting HF and LV systolic dysfunction who should be the primary MDT member and responsible for implementing and monitoring optimal GDMT [23, 80].

An evaluation for the cardiac resynchronization procedure (CRT) should be performed in patients for whom the indication exists according to the principles of the corresponding guidelines [72, 84].

If symptoms persist despite optimization of conventional therapy for heart failure, the choice of mechanical intervention on the mitral valve, with options using standard surgery or TEER, should be promptly considered to avoid further deterioration of left ventricular systolic function or cardiac remodeling.

### 6.2 Mechanical Intervention

Patients with chronic SMR often have a compromised prognosis [18, 39, 83, 84, 85] and for them, the optimal interventional approach is complex as evidenced by the analysis of Fig. 6. Given the difficulty of the framework to be examined, the role played by a multidisciplinary Heart Team in the decision-making is of primary consideration.

#### 6.2.1 The Work of the Heart Team

The Heart Team, which includes a specialist in heart failure management, should optimize guideline-oriented medical therapy (GDMT) and evaluate the different therapeutic paths: the indication for electrophysiological intervention, the indication for the catheter approach or standard surgery, considering risk vs benefits as well as the sequence of fulfillment. Given the paucity of multicenter RCTs to support a high rate for LOE, evidence that decisively supports the use of standard surgery remains limited.

In ESC guidelines for patients with severe SMR and indication for CABG operation or other cardiac surgery, the use of mitral valve surgery is recommended. The Heart Team has the task of evaluating the standard surgical approach by adapting the procedure very precisely to the clinical characteristics of each patient [39, 72, 83, 84, 85]. For patients who do not experience advanced left ventricular remodeling, the restrictive mitral annuloplasty with a complete rigid ring is recommended, leading to restoration of valve competence, improvement of symptoms, and resulting in reverse remodeling of the left ventricle [39, 83, 85]. As for the use of subvalvular techniques or chordal sparing valve replacement, these procedures may be considered for those patients in whom echocardiographic predictors tend to have a high risk of repair failure [40, 41, 86, 87, 88, 89, 90, 91, 92].

Although the use of valve replacement reduces the risk of recurrence of mitral regurgitation, however, this procedure is not associated with improved reverse left ventricular remodeling and does not lead to improved survival [42, 93]. The small number of multicenter RCTs have raised the problem of the limitation of indications for isolated mitral valve surgery in patients with severe SMR. This aspect is due to the high risk of negative side effects inherent to the procedure, high rates of recurrent mitral regurgitation, and the absence of demonstrated survival benefit occur in this population treated with standard surgery [41, 42, 91, 92]. One example that differs is represented by patients with severe SMR sustained by atrial fibrillation. This patient population usually has normal LVEF and left ventricular dilation is lower with smaller LV size. Since the main patho-anatomic feature is mitral annular dilation, which is the main mechanism of mitral regurgitation, this subpopulation responds effectively to RMA, often coupled with AF ablation. However solid evidence supporting this approach is still limited [84, 94] (Fig. 6).

**Fig. 7.**
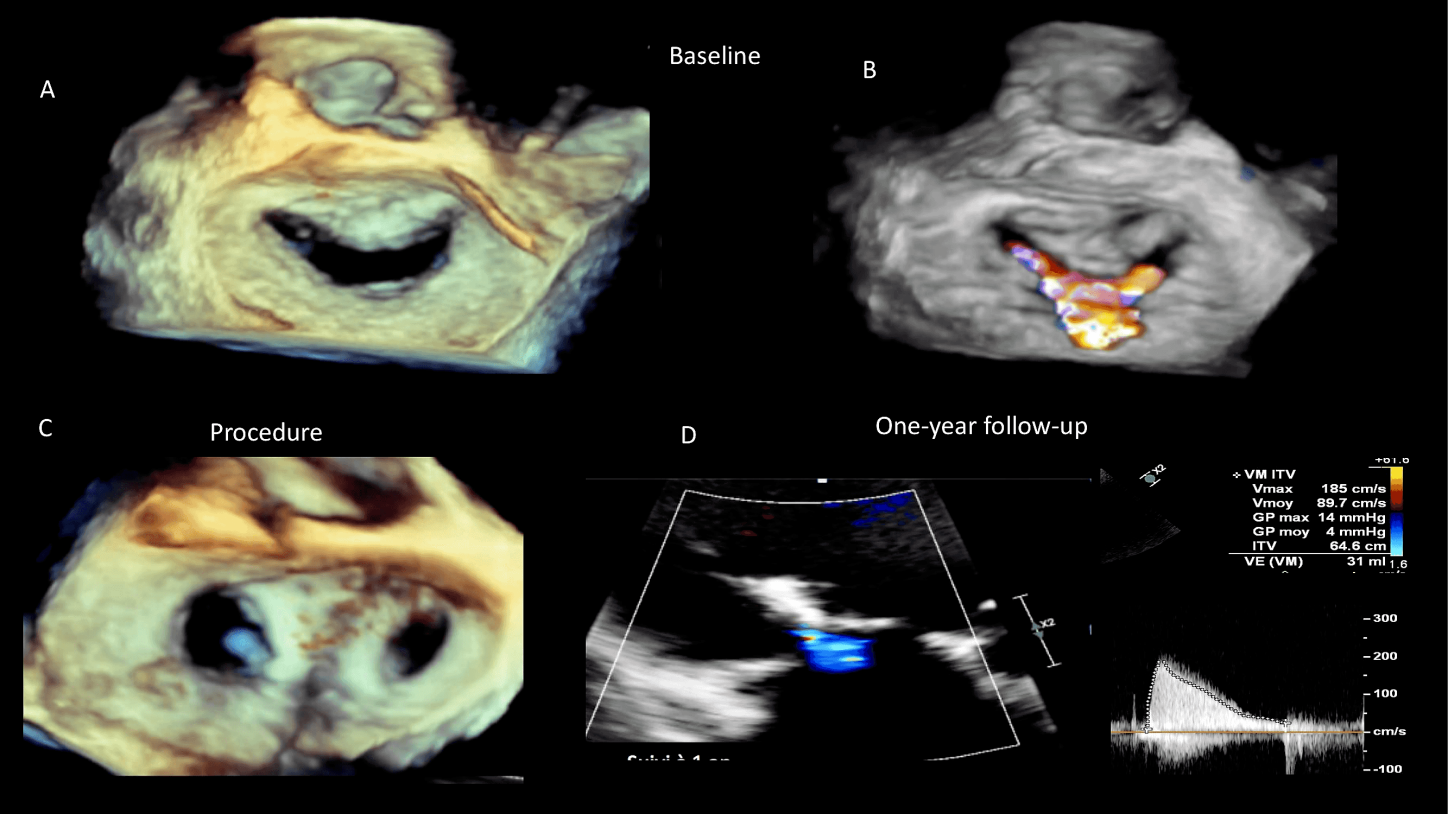
**TEER in patient with ischemic SMR**. 3D-TEE (A) and 3d-TEE color (B) en-face view showing central ischemic secondary MR. (C) 3D-TEE e-face view after a successful procedure with implantation of 2 central Mitraclips. (D) TTE 3-chamber view showing persistent good results at 1 year with residual mild MR and no hemodynamic stenosis (mean transmitral gradient at 4 mmHg).

The use of TEER with the MitraClip system has established itself as a minimally invasive approach, representing a further option of mechanical intervention for SMR. The two RCTs (COAPT and MITRA-FR) [23, 43, 44] that evaluated the safety and efficacy of TEER in patients with symptomatic heart failure and severe persistent SMR despite optimal medical treatment, for the evidence produced were considered by the Heart Teams for those patients judged ineligible or unsuitable to receive standard surgery (Fig. 6). The findings in the COAPT RCT with the three-year follow-up recorded that the procedure was safe in effectively reducing SMR [23, 38]. However, the data reported in the MITRA-FR study [43, 44], revealed that the use of MitraClip did not lead to any favorable impact on the primary endpoint of all-cause mortality or hospitalization for heart failure at 12 months and 2 years compared to GDMT alone. Regarding the COAPT study [23], evidence suggests that the use of MitraClip markedly reduced the primary endpoint of cumulative hospitalizations in patients requiring rehospitalization for heart failure. The study also demonstrated efficacy for several pre-specified secondary endpoints, including all causes of 2-year mortality. In Fig. 7 TEER was used in patient with ischemic SMR. 3D-TEE (Fig. 7A). At 1 year with residual mild MR and no hemodynamic stenosis were disclosed.

The two RCTs revealed conflicting results which generated a very intense discussion. These diverging results could be partially explained by the differences in study design that lead to the inhomogeneity of the enrolled patient population. In addition, investigators found as an important point of discussion the effect size of the trials, the echocardiographic assessment of the severity of SMR, and the use of optimal medical treatment. Patients enrolled in the COAPT study had higher SMR severity (EROA 41 ± 15 ${\mathrm{mm}}^{2}$ vs 31 ± 10 ${\mathrm{mm}}^{2}$) and less left ventricular dilation(mean indexed end-diastolic volume LV 101 ± 34 mL/$m^{2}$ vs. 135 ± 35 mL/$m^{2}$) compared to patients who were included in the MITRA-FR study. The divergence in results may be due to the increased severity of SMR in relation to left ventricular size (“disproportionate” mitral regurgitation) for patients who participated in the COAPT study. In fact, they were more likely to benefit from TEER in terms of reduced mortality and hospitalization for heart failure [95]. Thus, the differences reflect the need to undertake other studies.

Considering the results of the COAPT study, it appears that patients with severe SMR for whom TEER is recommended should comply with the COAPT inclusion criteria. They must receive optimal medical therapy and undergo scheduled checks by a specialist expert in heart failure as well as having the characteristics as similar as possible to those of the patients enrolled in the study. The optimization of the procedural result is an objective to be pursued which is in the interest to push for the TEER approach. Furthermore, it is important to clarify that the recommendation to receive a TEER can only apply to selected patients when the COAPT criteria are not met to improve symptoms and quality of life [96, 97]. In patients with less severe SMR (EROA <30 ${\mathrm{mm}}^{2}$) but with advanced left ventricular dilation/dysfunction, doubts persist about the prognostic benefit after MitraClip which remains unproven. The consideration for the use of the device is also to be avoided for patients with end-stage left ventricular and/or right ventricular failure and for whom myocardial revascularization with a different approach, PCI or CABG, is not indicated. These patients benefit most when they receive a heart transplant or when a left ventricular assist bridge device is used. The point that remains indisputable is that the surgery of the mitral valve is generally not a valid option in patients presenting with LVEF <15% [39, 74, 85].

The management of moderate ischemic SMR in patients undergoing CABG remains controversial and is frequently debated [36, 98]. The best option for these patients is to consider surgery if they have good myocardial viability and if the comorbidity score is low. In patients diagnosed with exercise-induced dyspnea and a sharp increase in the severity of mitral regurgitation and SPAP, combined surgery is the most suitable.

Advances in technology have proposed novel transcatheter mitral valve repair systems other than TEER that complement transcatheter mitral valve replacement devices. All of these devices are currently the subject of intense investigations and may hinge on clinical data that are still limited [99].

#### 6.2.2 Recommendations from ACC/AHA Guidelines

In ACC/AHA guidelines [2] for patients who had chronic severe SMR (Stage D) from depressed LV systolic dysfunction (LVEF <50%) and with persistent severe symptoms (NYHA class II, III, or IV) and on optimal GDMT for HF, TEER is recommended. The use of TEER is reasonable for patients who have favorable anatomy that is defined on TEE and presenting with LVEF between 20% and 50%, LVESD ≤70 mm, and pulmonary artery systolic pressure ≤70 mmHg (318,338–344) (COR 2a/LOE B-R) [2] (Fig. 6).

Mitral valve surgery is reasonable in patients who require CABG operation for the treatment of myocardial ischemia (COR 2a/LOE B-NR) [41, 100, 101, 102, 103, 104]. Mitral valve surgery is also considered for patients in stage D who have severe persistent symptoms including in NYHA class III or IV despite optimal GDMT for HF and who were managed with therapy for associated AF or other comorbidities. Patients with AF and annular dilatation generally experience a higher LVEF (≥50%) (COR 2b/LOE B-NR) [104, 105, 106]. In patients with lower systolic dysfunction (LVEF <50%) the use of chordal-sparing mitral valve replacement may be more suitable compared to RMA (COR 2b/LOE B-NR) [18, 36, 90, 93, 103] (Fig. 6).

In the ACC/AHA guidelines, we have noted five recommendations of which 2 recommendations are included in the class of recommendation (COR2a) which states the usefulness of the procedure but with moderate benefit versus risk. 3 Recommendations are graded as COR 2b which indicates the usefulness of the procedure is related to a weak benefit versus risk. We disclosed no recommendations graded as COR 1a which indicates a strong benefit versus risk. None of these are included in LOE A which is supported by a high level of evidence from 1 or more RCTs, meta-analyses of high-quality RCTs, and 1 or more RCTs confirmed by high-quality registry studies. In contrast, all 5 recommendations have an LOE BR or B-NR which are influenced by the moderate quality of 1 or more RCTs and meta-analysis or moderate quality of well-performed non-randomized studies, observational studies, or registry studies including meta-analysis of such studies [2] (Fig. 6).

From the available scientific literature, it is difficult to state which type of intervention is better because there are no RCTs (such as the PARTNER RCT) designed on a large number of enrolled patients that include candidates to receive TEER, mitral valve replacement, or mitral valve repair with or without a subvalvular procedure. For example, in the evaluation of the ACC/AHA guidelines regarding TEER we have 2 RCTs, MITRA Fr [43, 44] and COAPT [23], 1 comparison analysis between MITRA Fr and COAPT [107], 1 observational study with follow up at 1 year after the use of the Mitraclip procedure [108], the pivotal small observational study reporting the comparison between TEER and standard surgery [109] and the analysis of the new pathophysiological picture of the proportionate/disproportionate condition for SMR [95]. The latest report supports the use of TEER in patients enrolled in the COAPT RCT study. Note that the study reporting the MITRA Fr result at 2 years of follow-up was excluded and we have only 1 study published in 2014 that compared the standard surgical procedure with TEER in high-risk patients [44] (Fig. 6).

In the recommendation for the use of TEER (COR 2a LOE B-R) we do not find any specific indication for the treatment of myocardial ischaemia [2]. In the COAPT RCT study although we can observe 60.9% of patients with ischemic cardiomyopathy, however only 43% and 40% of patients received PCI or CABG, respectively [23]. Concerns about left ventricular remodeling in the presence of extensive scar tissue formation after myocardial infarction or protracted myocardial ischaemia with wall motion abnormalities is a major problem in patients with secondary mitral insufficiency included in the Carpentier type IIIb classification. The presence of diffuse coronary heart disease not adequately treated can be the cause of the evolution of the SMR due to ischemic cardiomyopathy towards a picture similar to that of the nonischemic cardiomyopathy [110, 111]. In these cases, SMR can also cause central mitral regurgitation and if as mentioned precedently, there are global wall motion abnormalities from multivessel coronary disease leading to equal lateral displacement of both papillary muscles similar to that seen in nonischemic cardiomyopathy [41, 86, 87, 92].

**Fig. 8.**
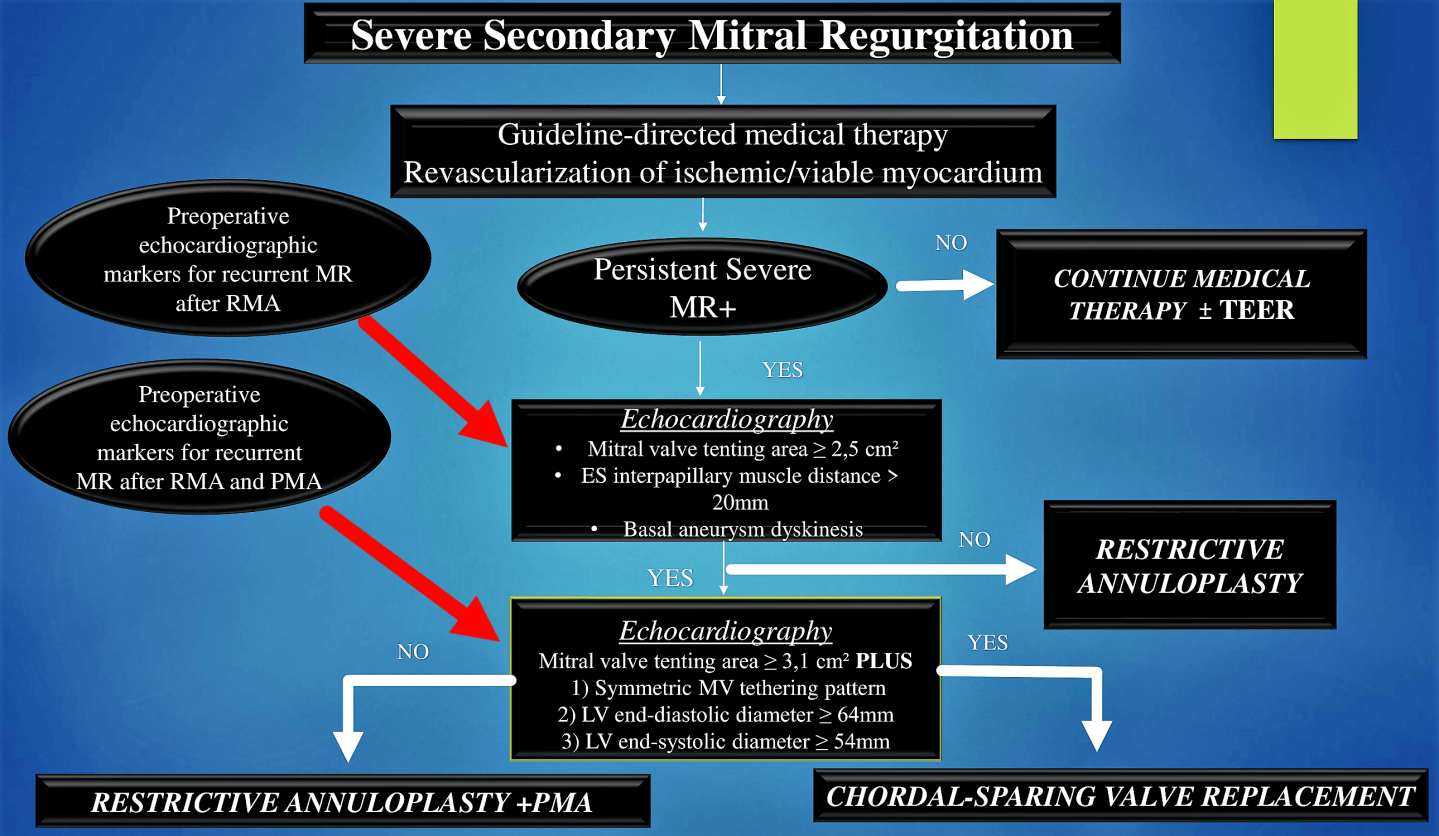
**Depicts the treatment algorithm for patients with severe secondary MR undergoing MV surgery**. Patients with severe secondary MR receive either isolated RMA or RMA combined with subannular repair and coronary artery bypass operation. Preoperative echocardiographic markers for recurrent MR evaluate the result of MV repair after undersized restrictive ring annuloplasty (Up red arrow). Preoperative echocardiographic markers for recurrent MR evaluate the result of MV repair after the use of combined of RMA and PMA (down red arrow). Abbreviations: PMA, papillary muscle approximation. Others abbreviation in previous figures. From Petrus AHJ, *et al*. [39]; Harmel EK, *et al*. [40]; Nappi F, *et al*. [41, 86, 87, 88, 89, 90, 91, 92]; Acker MA, *et al*. [42]; Michler RE, *et al*. [98].

In our previous study, we disclosed that in secondary mitral valve regurgitation the change of geometric LV shape with distortion of the normal spatial relationships of the elements of the MV can be normalized with the recovery of anteroposterior annular dilation, tenting area, and interpapillary muscle distance [86, 87]. In severely dilated left ventricular chambers with LVEDD ≥65 mm and with LVEF between 20% and 50% the use of TEER did not improve left ventricular remodeling because the tethering exerted on the leaflets with an apical tenting of the anterior leaflet could not be improved [41, 43, 44, 88, 90, 95]. Conversely, in patients with left Venticualar End Diastolic Dimension (LVEDD) ≤5 mm and LVEF >40%, the use of TEER has proven efficacy in reducing rehospitalization rates for heart failure and MR recurrence [23, 41, 95] (Fig. 8, Ref. [39, 40, 41, 42, 86, 87, 88, 89, 90, 91, 92, 98]).

## 7. Conclusions

The Current guidelines by the ESC and AHA/ACC raise some important questions regarding the current management of secondary mitral regurgitation. However, most of these are not backed by high level of evidence which should be the next avenue to consider. The role of echocardiography and other imaging modalities are important for classifying severity. Decision-making regarding the modality of care should be led by members of the heart team.
